# Person-centredness in the care of older adults: a systematic review of questionnaire-based scales and their measurement properties

**DOI:** 10.1186/s12877-016-0229-y

**Published:** 2016-03-07

**Authors:** Mark Wilberforce, David Challis, Linda Davies, Michael P. Kelly, Chris Roberts, Nik Loynes

**Affiliations:** Personal Social Services Research Unit, Precinct Centre, Crawford House, University of Manchester, Oxford Road, Manchester, M13 9PL UK; Institute of Population Health, Jean McFarlane Building, University of Manchester, Oxford Road, Manchester, M13 9PL UK; Institute of Public Health, Forvie Site, Cambridge Biomedical Campus, University of Cambridge, Cambridge, CB2 0SR UK

**Keywords:** Person-centred care, Patient-centred medicine, Systematic review, Measurement, Psychometrics, Community care, Older people, COSMIN

## Abstract

**Background:**

Person-centredness is promoted as a central feature of the long-term care of older adults. Measures are needed to assist researchers, service planners and regulators in assessing this feature of quality. However, no systematic review exists to identify potential instruments and to provide a critical appraisal of their measurement properties.

**Method:**

A systematic review of measures of person-centredness was undertaken. Inclusion criteria restricted references to multi-item instruments designed for older adult services, or otherwise with measurement properties tested in an older adult population. A two-stage critical appraisal was conducted. First, the methodological quality of included references was assessed using the COSMIN toolkit. Second, seven measurement properties were rated using widely-recognised thresholds of acceptability. These results were then synthesised to provide an overall appraisal of the strength of evidence for each measurement property for each instrument.

**Results:**

Eleven measures tested in 22 references were included. Six instruments were designed principally for use in long-stay residential facilities, and four were for ambulatory hospital or clinic-based services. Only one measure was designed mainly for completion by users of home care services. No measure could be assessed across all seven measurement properties. Despite some instruments having promising measurement properties, this was consistently undermined by the poor methodological quality underpinning them. Testing of hypotheses to support construct validity was of particularly low quality, whilst measurement error was rarely assessed. Two measures were identified as having been the subject of the most rigorous testing.

**Conclusion:**

The review is unable to unequivocally recommend any measures of person-centredness for use in older adult care. Researchers are advised to improve methodological rigour when testing instruments. Efforts may be best focused on testing a narrower range of measurement properties but to a higher standard, and ensuring that translations to new languages are resisted until strong measurement properties are demonstrated in the original tongue. Limitations of the review include inevitable semantic and conceptual challenges involved in defining ‘person-centredness’.

The review protocol was registered with PROSPERO (ref: CRD42014005935).

**Electronic supplementary material:**

The online version of this article (doi:10.1186/s12877-016-0229-y) contains supplementary material, which is available to authorized users.

## Background

‘Person-centredness’ is internationally regarded by many as a foundation for modern health and social care services [1–3], with the World Health Organization recently calling for a ‘fundamental paradigm shift’ in strategy and delivery in accordance with its principles [4]. It has widespread appeal as a philosophy of care that emphasizes the need for services to be responsive to individual needs, and promotes the rights of recipients in achieving a greater influence over decisions that affect them [5–7]. Tracing its origins back to the 1950s, person-centredness can draw upon a spectrum of well-established conceptual frameworks, including personhood; normalisation; the social model of disability; citizenship; and new public management. In England, person-centredness is championed throughout the care system, from the National Health Service Constitution, through legislative programmes and individual policy initiatives, national clinical standards, regulation of care quality, indicators of performance and, ultimately, front-line practice [8–10]. That person-centredness has come to hold such a prominent position in the care system is no accident. In addition to ethical arguments based on human rights and public service accountability, evidence suggests that it is strongly associated with service satisfaction; is linked with better engagement with, and adherence to, treatment plans; and is broadly associated with improved health and quality-of-life outcomes [11–13].

Despite attaining such prominent status, `person-centredness' is notoriously difficult to define and conceptualise. Reviews commonly regard person-centredness as a composite [1, 7, 14] in combining care attributes that themselves are independently recognized components of quality. Different traditions of ‘centredness’ can be identified within the literature, using varied prefixes (eg ‘patient’, ‘client’, or ‘consumer’) with each giving different emphasis to its necessary and sufficient attributes. Nevertheless three themes are common to each, together forming an operational definition of person-centredness used in this review. First, it gives primacy to *understanding the person* and their unique interpretation and experience of illness or disability, in particular by taking a holistic view through recognition of psycho-social factors beyond presenting symptoms [12]. Second, service user *empowerment in decision-making* has been described as the ‘pinnacle’ of person-centredness [15], with greater delegation of control over choices to the service user, guided by a practitioner through appropriate information sharing [16]. Third, the importance of *relationships in care* and treatment is prioritized, since positive and respectful interpersonal exchanges and the development of trust built on continuity and coordination in care are viewed as therapeutic vehicles to successful support [2].

The importance of person-centredness for older people with long-term conditions may be at least as great as for other patient groups. The prevalence of multi-morbidity and long-term health problems increases with age, requiring many older adults to draw upon a wider range of support often from multiple care professionals and providers, and so increasing the risk of fragmented care relationships. Further, older people may prioritise the affective characteristics of the care exchange as much as the achievement of specific outcomes [17, 18]. This may reflect the value placed by older people on maintaining personal identity and usual routines in the context of cognitive or physical decline which can, in part, be achieved through positive care interactions and attention to the whole person [19]. Older people may also prioritise different facets of person-centredness, or require them to be achieved in a different manner. For example, preferences for autonomy and engagement in decision-making vary between cross-sections of younger and older adults [20].

Given conceptual ambiguity, and potential subtle differences in priorities and articulation amongst older people, it is unsurprising that no clear set of measures is available to assist service planners, regulators or researchers in assessing person-centred qualities. Yet recent international appeals to improve and standardise approaches to measurement have brought renewed attention to the need for appropriate instruments [4]. A Cochrane Review relating to clinical consultations [21] found that none of the included studies used direct measurement of person-centredness, precluding an understanding of cause-effect pathways, and adding to claims that current scales are either not fit for purpose or inconsistently used [7, 11, 12, 22]. Instead, broad satisfaction surveys are commonly adopted, which routinely identify positive experiences amongst older people but which are doubted both conceptually and empirically [23, 24].

No systematic review of measures of person-centredness relevant to the long-term care of older adults has yet been conducted. Most importantly, narrative descriptions of available measures [16, 25] have made reference to measurement properties *without* critically appraising the quality of research underpinning them. As in any research field, the acceptance of empirically-derived estimates without critical appraisal undermines the evidence-base [26]. The purpose of this review is to address this gap. Specifically, the review aimed to identify, describe and critically appraise measures of person-centredness relevant to the long-term care of older people.

## Methods

### Search strategy

The review protocol was registered with PROSPERO (ref: CRD42014005935). The principal search sought to identify three concepts: “person-centred”; “older people’s services” and “quality measures”. With respect to the former, search terms also included the prefixes (“patient”, “consumer”, “client”) and suffixes (“led”, “oriented”, “directed”) to ‘centredness’. Further, the search included “individualized” and “personalized” alternatives, and both UK and US spellings. For older people’s services, variants were “older people”, “older person”, “elder*”, “old aged”, “geriatr*” and “senior*”. To identify quality measures, the search terms were extended to “measure*”, “questionnaire”, “instrument”, “scale”, “index”, “schedule”, “inventory” and “psychometrics”. An example search strategy is included as a supplementary file.

Searches were undertaken in Pubmed, CINAHL, Web of Science, PsycINFO, ASSIA, and Social Science Abstracts databases. The search strategy was piloted and refined through discussion between all authors. This search was complemented by a manual review of the bibliographies and measures of person-centredness identified in other reviews, and contact with a leading author in the field. Finally, those measures included in the review were then the subject of an additional search for other references testing the same measures (for example, in other service settings, or testing other psychometric properties).

### Study selection

Once duplicates were removed, a two-stage sifting process was undertaken. First, one reviewer (MW) screened the titles and abstracts of all citations, seeking to identify those of relevance to the review. All excluded references were screened by a second reviewer (NL). Any ambiguous citations were retained, in addition to those where an abstract was missing. At the second stage, all full articles of the remaining references were obtained and reviewed separately by two authors (MW, DC), achieving an 88 % agreement. Disagreements were resolved by discussion and final consensus.

Five criteria guided the selection of articles, which were refined during the process of piloting (with PROSPERO updated accordingly). First, included instruments were questionnaire-based, and thus excluded measures using direct observation or recordings of care interactions. Second, references needed to report at least one measurement property of a multi-item scale, defined as those assessed by COSMIN (COnsensus-based Standards for the selection of health Measurement INstruments) guidelines [27], detailed below. It was not required that authors explicitly stated their intent to establish a measurement property as a research aim, only that information of potential value in doing so was reported. Third, instruments were included if the authors provided evidence of an intent to measure person-centredness, such as through the stated aim of the measure. Where this was not evident, the theoretical framework, background and rationale for the measures were explored for reference to forms of “centredness” as a guiding principle to the instrument’s development. Measures were excluded if no such evidence could be discerned. Through this criterion, generic quality measures and satisfaction scales were excluded. Fourth, the review is also restricted to measures tested with an older adult population, or in older people’s services, defined as those being exclusively (eg by referral criteria, such as age restrictions) or predominantly (eg by nature of service, such as dementia care) used by older adults. Where this was not clear, the characteristics of the sample used in testing the measure were inspected. Finally, measures relating to short-term services (such as emergency medicine) were excluded.

**Fig. 1:**
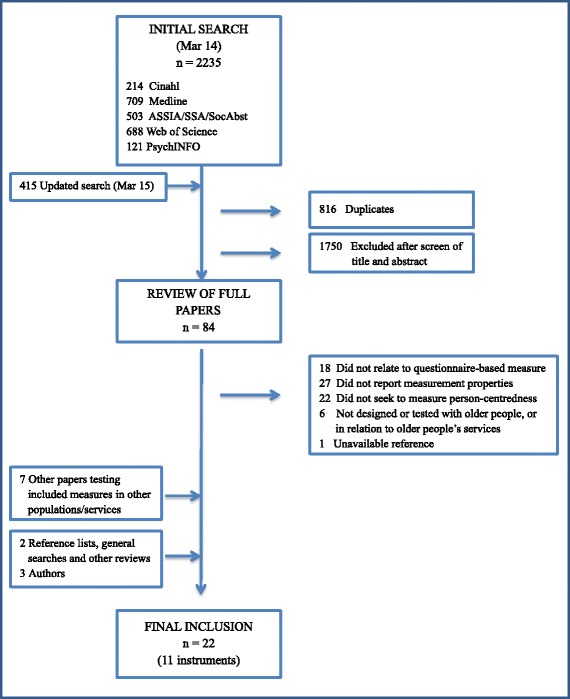
Search and selection process

The initial electronic search was undertaken in March 2014, and updated in April 2015. Of 2650 references included in the electronic searches, 84 were retained as potential inclusions and read in full (Fig. 1). Twelve other references were found through other searches. The review is based on 11 instruments, reported in 22 separate references.

### Data extraction and critical appraisal

Information relating to the characteristics and aims of the measure; its development and underpinning framework; the domains and items included; the service setting and mechanism of application; and measurement properties were extracted from each reference. Critical appraisal entailed a three-step procedure, informed by the COSMIN framework [27]. First, the methodological quality of the studies was assessed using the COSMIN checklist. This process generated a separate rating (excellent/good/fair/poor) for each of seven measurement properties, where estimated, in each reference. Second, the estimated measurement properties were assessed against established thresholds of acceptability (see Table 1). Finally, for each instrument, these assessments were combined to provide an overall rating of the strength of evidence for each measurement property, using a scale adapted by Schellingerhout et al. [28] from the Cochrane Back Review Group (Table 2). Quality appraisal was piloted by four authors with one reference, and two authors then independently reviewed a further five references. From that point data extraction was by one author, and corroborated by the second. A completed PRISMA checklist is included as Additional file 1.

**Table 1:** Criteria for assessing measurement properties (adapted from Schellingerhout et al. [28])

Measurement property	Rating	Criteria
Reliability		
*Internal consistency*	+	Cronbach alpha > =0.70 and < 0.95
	-	Cronbach alpha <0.70 or > =0.95
	?	Not available, or scale/subscale not established as unidimensional
*Reliability*	+	Intraclass Correlation Coefficient (ICC) > = 0.70 or Pearson’s Correlation Coefficient (r) > =0.80
	-	ICC <0.70 or r <0.80
*Measurement error*	+	Minimal important change (MIC) > Smallest Detectable Change (SDC)
	-	MIC < = SDC
	?	MIC not established
Validity		
*Content validity*	+	Assessed in target population that items are a complete representation of concept under measurement and that all items are relevant.
	-	Questionnaire is incomplete or contains irrelevant items
	?	Not available, or not assessed in target population
*Structural validity*	+	Factors explain 50 % of variance
	-	Factors explain less than 50 % of variance
	?	Explained variance not presented
*Hypothesis testing*	+	Correlation with instruments measuring related constructs is higher than unrelated constructs, AND either (correlation with instrument measuring related construct > =0.50 OR at least 75 % of hypotheses conform to expectations).
	-	Correlation with instruments measuring unrelated constructs higher than related constructs OR correlation with instrument measuring related construct <0.50 OR fewer than 75 % of hypotheses conform to expectations.
	?	Correlations only with unrelated constructs, or hypotheses not sufficiently-well specified.
*Cross-cultural validity*	+	Original factor structure confirmed OR no differential item functioning
	-	Does not conform to original factor structure, or important differential item functioning observed
	?	Factor analysis or differential item functioning not presented

**Table 2:** Quality synthesis

Level	Rating	Description
Strong	+++ (−−−)	Consistent positive (negative) ratings derived from multiple studies of good quality, or in one study of excellent quality
Moderate	++ (−−)	Consistent positive (negative) ratings in multiple studies of fair quality, or in one study of good quality
Limited	+ (−)	Positive (negative) rating in one study of fair quality
Conflicting	+/−	Conflicting results
Unknown	?	Only studies of poor quality

## Results

Of the 11 instruments included in the review, four stood out as having been the subject of tests of measurement properties in three or more studies, and together accounted for over half of the 22 references: the Individualised Care Instrument (ICI) [29–31]; Person-Centred Care Assessment Tool (P-CAT) [32–35]; the Person-centred Climate Questionnaire (PCQ) (comprising both staff [36, 37] and patient) [38] versions); and the Client-Centred Care Questionnaire (CCCQ) [39–42]. The remaining seven instruments had not been as extensively tested in an older adult population, although the Individualized Care Scale – Nurse (ICS-N) [43] and Measures of Processes of Care – Adult (MPOC-A) [44] were more widely used outside specialist old-age services. An ‘Untitled’ measure was also included in this review [45], but differed from others since establishing measurement properties was not the main focus of the associated reference.

To assist in synthesis, measures were organised according to whether they were specifically designed for application in older adult services (*n* = 6, hereafter ‘specific’), or else were originally designed for other/generic services, but had since been applied to older adult services, or in a predominantly older sample (*n* = 5, hereafter ‘generic’). Table 3 illustrates that all except one of the specific instruments (Patient-Centered Family Focused Care (PCFC) [46]) were initially designed for completion by practitioners, whilst the pattern was largely reversed for generic measures. This tallies with the service settings of the former; predominantly relating to long-stay care designed for people with dementia, thus likely to preclude self-completed questionnaires. Six instruments were designed primarily for use in residential or long-stay nursing care settings (ICI, P-CAT, Person-Directed Care (PDC) [47], 'Untitled', PCQ, ICS-N); four were designed for ambulatory hospital- or clinic-based services (PCFC, Person-Centred Health Care for Older Adults (PCHC) [48], MPOC-A, Client-Centred Rehabilitation Questionnaire (CCRQ) [49]) and with just one measure (CCCQ) designed explicitly for home-based services. The measures drew on a range of different traditions of ‘centredness’ to inform their development, such as a Kitwoodian analysis [19] of respect for personhood in dementia (P-CAT, 'Untitled'), and client-centredness in rehabilitation (CCRQ). The origins of two other specific measures (PCHC, PDC) lay with policy-makers rather than clinicians and academia: for example, the PDC measure supported a programme to improve the standing and attractiveness of work in the long-term care of older adults in Oregan [47]. Just three measures (ICI, CCCQ, CCRQ) sourced items empirically from primary exploratory fieldwork with service users, using a range of qualitative techniques (observation of care exchanges; qualitative semi-structured interviews; and focus groups). The CCRQ was unique in undertaking formal cognitive interviews as a mechanism for testing with service users how items were interpreted and how responses formulated [49].

**Table 3:** Overview of included instruments

Name of instrument	Year of first reference in review	Respondent	Conceptual origins	Service/setting context	Method for item generation	Validation methods
*Specific measures: instruments originally designed for use with older adults, or in relation to older adult services*						
Individualised Care Instrument (ICI)	2007 [29]	Care worker	Individualised nursing care in long-term institutions for people with dementia.	Residential and nursing home settings; long-stay hospital wards; sheltered housing; home services and other long-stay care facilities.	Observation of care interactions, and literature.	Expert panel
Person-Centred Care Assessment Tool (P-CAT)	2010 [32]	Practitioners	Personhood in dementia and subjective experiences of illness.	Long-term aged care and residential care settings	Literature	Expert panel and focus group of service users
Patient-Centered Family-Focused Care (PCFC)	2007 [46]	Service user/family carer	Palliative care literature integrating ‘whole person’ perspectives with family-centredness	Frail elders using veteran ambulatory care centres	Theory, literature and existing instrumentation	Not specified
Person-Centred Health Care for Older Adults (PCHC)	2013 [48]	Multiple staff groups	Policy-driven conceptualisation of person-centredness in hospital settings	Hospital wards, rehabilitation and continence clinics	Research team and literature	Expert panel and focus group of service users
Person-Directed Care Measure (PDC)	2008 [47]	Multiple staff groups	Policy-driven origins: measure designed to evaluate local person-centred care initiative with aim of improving care relationships and job satisfaction	Residential care, assisted living and home care settings.	Research team, practitioners and literature.	With practitioners
‘Untitled’	2013 [45]	Nurses	Personhood in dementia	Long-term geriatric wards	Interviews with practitioners, expert opinion and literature	None presented
*Generic measures: instruments initially designed for wider services*						
Person-centred Climate Questionnaire (PCQ)	2012 [37]	Service user and staff versions	Person-centredness in care environment	Nursing homes, dementia care wards and other long-term care facilities	Theory and literature	Expert panel
Client-Centred Care Questionnaire (CCCQ)	2006 [39]	Service user	Client-centredness in home-based nursing care for people with long-term conditions	Home care services; long-term hospital wards	Qualitative interviews with service users	Expert panel
Individualised Care Scale – Nurse (ICS-N)	2012 [43]	Nurses	Individualised care as an application of interactional models of nursing.	Long-term care wards	Literature	Expert panel
Measures of Processes of Care – Adult (MPOC-A)	2010 [44]	Service user/family carer	Client and family-centred care in paediatric medicine	Community orthopaedic services	Existing instruments	Research team
Client-Centred Rehabilitation Questionnaire (CCRQ]	2006 [49]	Service user	Client-centred occupational therapy in rehabilitation services, drawing on principles that promote autonomy, client strengths, choice and partnership.	Ward-based rehabilitation program	Focus groups with service users	Cognitive interviews with service users

Eight instruments (see Table 4) were multidimensional and formed distinct subscales, typically identified through factor analyses, enabling an assessment of how well the three broad themes of person-centredness (outlined above) were represented. Items relating to *'understanding the person*' formed a distinct subscale of the ICI and PDC (both labelled 'knowing the person'); PCHC ('getting to know the individual'); and the ICS-N (comprising separate subscales assessing how well practitioners attended to the personal experiences and interpretation of their current ‘clinical situation’; and a second focused on understanding the patient’s wider ‘life situation’). Items relating to *‘empowerment in decision-making’* were distinct in the ICI and PDC (both labelled 'autonomy'); PCHC ('involvement in care planning'); the ICS-N ('decisional control over care'); MPOC-A ('enabling and partnership') and CCRQ ('decision making'). Furthermore, the MPOC-A and CCRQ additionally included subscales directed at the quality of information-sharing in supporting decisions; and the P-CAT included a subscale assessing how care decisions are tailored to the individual. Finally, features of *'relationships in care'* were evident through items in the ICI ('communication with residents'); PCHC ('supporting relations'); MPOC-A ('respectful and supportive care') and CCRQ ('emotional support'). The extent of continuity and coordination in care was also a feature of the PCHC, MPOC-A and CCRQ. Whilst the labels of the PCQ subscales do not obviously correspond with the themes of person-centredness used in this paper, a closer inspection of individual items finds clear resonance. For example, within ‘a climate of safety’ are several items relating to caregiver interpersonal and relationship skills.

**Table 4:** Attributes of person-centredness

Name of measure	No. items (scales)	Attributes
*Specific measures*		
ICI	22 (4)	Knowing the person (6 items); Autonomy (8 items); Communication (Staff/Resident) (3 items); Communication (Staff/Staff) (5 items).
P-CAT	13 (3)	Extent of personalising care (7 items); Amount of organizational support (4 items); Degree of environmental accessibility: (2 items).
PCFC	8 (1)	Unidimensional scale
PCHC	31 (8)	Involvement in care planning (4 items); Finding out goals (2 items); Supportive working environment (7 items); Coordinated contact (4 items); Meeting practical needs (4 items); Meeting communication needs (4 items); Getting to know the individual (3 items); Attitudes towards person-centred care (3 items).
PDC	35 (5)	Knowing the person (7 items); Comfort care (8 items); Autonomy (7 items); Personhood (7 items); Support relations (6 items).
‘Untitled’	8 (1)	Unidimensional scale
*Generic measures*		
PCQ (Staff version)	14 (3)	A climate of safety (6 items); A climate of everydayness (4 items); A climate of community (4 items)
PCQ (Patient version)	17 (3)	A climate of safety (10 items); A climate of everydayness (4 items); A climate of hospitality (3 items)
CCCQ	15 (1)	Unidimensional scale
ICS-N	17 (3)	Clinical situation (7 items); Personal life situation (4 items); Decisional control over care (6 items)
MPOC-A	34 (4)	Enabling and partnership (9 items); Providing general/specific information (10 items); Coordinated and comprehensive care (9 items); Respectful and supportive care (6 items)
CCRQ	30 (7)	Decision-making (5 items); Information-sharing (4 items); [Involvement in] Outcome evaluation (4 items); Family involvement (5 items); Emotional support (4 items); Physical comfort (4 items); Continuity in care (4 items).

### Quality appraisal and synthesis

Table 5 presents an assessment of the methodological quality of the research upon which measurement properties were estimated. Where a measurement property was not estimated, the table cell is blank. All references attempted an assessment of internal consistency, which were typically well performed [34–48, 37, 39, 42] particularly where supported by large sample sizes. Tests of structural validity were of more diverse quality, with some references missing opportunities for confirmatory factor analyses to more firmly establish structures identified in earlier exploratory work [35, 48, 43, 37, 43]. Test-retest reliability was performed in 10 references, but was not well conducted overall because of inadequate sample sizes [29, 32, 34, 35, 45, 37, 44] or a sub-optimal choice of correlation coefficient (e.g. Pearson’s *r* chosen over intra-class correlation coefficient [37]). Content validity was also typically poorly conducted since it was rarely assessed if the items comprehensively spanned the person-centredness construct as defined by the authors [29, 46, 45, 37–39]. Hypothesis testing also tended to be inadequate, since analysis with the potential to support concurrent validity was often stymied by failing to describe the comparison instrument in sufficient detail [33, 39, 42]. Further, anticipated directions and magnitudes of associations in hypotheses testing were rarely specified with clarity [30, 36–44, 49]. For example, Cott et al. [49] hypothesised that differences would be observed in CCRQ scores between different participating institutions in their study, without being sufficiently precise in their expectations. Further, they gave weight to significant differences from a large battery of statistical testing susceptible to Type I error. No measures assessed validity against a recognised ‘gold standard’.

**Table 5:** Methodological quality for studies across seven measurement properties

Measure	Study	Internal consistency	Reliability	Measurement error	Content validity	Structural validity	Hypothesis testing	Cross-cultural
*Specific measures*								
ICI	Chappell et al. [29]	Poor	Fair		Poor	Poor	Fair	
	Charalambous et al. [30]	Fair					Fair	Poor
	O’Rourke et al. [31]	Good				Good		
P-CAT	Edvardsson et al. [32]	Good	Poor		Good	Good		
	Zhong & Lou [33]	Fair				Fair	Poor	Poor
	Sjogren et al. [34]	Excellent	Fair			Excellent		Poor
	Rokstad et al. [35]	Excellent	Fair			Good		Poor
PCFC	Rose et al. [46]	Excellent			Poor	Excellent		
PCHC	Dow et al. [48]	Excellent			Excellent	Good		
PDC	White et al. [47]	Fair			Good	Fair		
	Sullivan et al. [52]	Fair			Poor			
‘Untitled’	Terada et al. [45]	Poor	Poor		Poor			
*Generic measures*								
PCQ (Staff version)	Edvardsson et al. [36]	Fair					Fair	
	Bergland et al. [37]	Excellent	Fair		Poor	Good	Poor	Fair
PCQ (Patient version)	Bergland et al. [38]	Fair	Fair		Poor		Poor	Fair
MPOCA	Bamm et al. [44]	Poor	Fair		Poor		Fair	
CCCQ	de Witte et al. [39]	Excellent			Poor	Fair	Poor	
	Bruus et al. [40]	Poor						Poor
	Bosman et al. [41]	Fair					Poor	
	Muntinga et al. [42]	Excellent	Excellent	Excellent		Excellent	Poor	
ICS-N	Suhonen et al. [43]	Fair				Fair		
CCRQ	Cott et al. [49]	Poor	Good		Excellent		Poor	

Empty cells indicate the property was not assessed in the reference

The measurement property ratings for each reference are presented in Table 6 using thresholds outlined above (Table 1). For measures with multiple subscales, a positive (or negative) rating required that *every* estimate meet (or fail) the threshold; where this was not attained an undetermined rating was given, although the number of estimates meeting the threshold for a positive result is provided in parentheses. Thus, internal consistency was most often 'undetermined’ because not all subscales met the requisite Cronbach alpha threshold, although each contained at least one subscale achieving this criterion. Test-retest reliability was mostly rated positively. Content validity was ‘undetermined’ in seven of the applications, including all of the generic measures [36–49] because the instruments did not incorporate specific validation in the older adult populations they were now being implemented within (a minimum requirement for any rating to be given on this domain). Three references testing hypotheses received a negative rating [30, 33, 42] and only one rated positively [39]. No applications of cross-cultural validity could be given a determined rating since they all failed the minimum standards established in Table 1.

**Table 6:** Rating of measurement properties against thresholds

Measure	Study	Internal consistency	Reliability	Measurement error	Content validity	Structural validity	Hypothesis testing	Cross-cultural
*Specific measures*								
ICI	Chappell et al. [29]	? ^(3/4)^	? ^(1/4)^		?	-	?	
	Charalambous et al. [30]	? ^(3/4)^					-	
	O’Rourke et al. [31]	? ^(7/8)^				+		
P-CAT	Edvardsson et al. [32]	? ^(3/4)^	? ^(1/4)^		+	+		
	Zhong & Lou [33]	? ^(1/4)^				?	-	?
	Sjogren et al. [34]	+	? ^(1/3)^			-		?
	Rokstad et al. [35]	+	+			-		?
PCFC	Rose et al. [46]	+			?	?		
PCHC	Dow et al. [48]	? ^(4/8)^			+	+		
PDC	White et al. [47]	+			+	+		
	Sullivan et al. [52]	+			+			
‘Untitled’	Terada et al. [45]	?	+		?			
*Generic measures*								
PCQ (Staff version)	Edvardsson et al. [36]	+					?	
	Bergland et al. [37]	+	-		?	+	?	?
PCQ (Patient version)	Bergland et al. [38]	?	+		?		?	?
MPOCA	Bamm et al. [44]	?	+		?		?	
CCCQ	de Witte et al. [39]	+			?	+	+	
	Bruus et al. [40]	?						?
	Bosman et al. [41]	+					?	
	Muntinga et al. [42]	?	+	?		+	-	
ICS-N	Suhonen et al. [43]	+				?		
CCRQ	Cott et al. [49]	?	+		+		?	

‘+’ indicates that the threshold was met; ‘-‘indicates that the threshold was failed’ ‘?’ indicates that a rating could not be determined from the results presented. Where results are inconsistent across subscales, a ‘?’ rating is given. Parentheses then how many of the subscales met the relevant thresholds. Empty cells indicate the property was not assessed in the reference

Finally, Table 7 presents a quality synthesis for each measure by combining the information from Tables 5 and 6 above. Due to missing measurement properties the table has only 51 populated cells (33 being empty). Further, fewer than half (*n* = 24) of the populated cells could be assigned a definitive (positive or negative) rating, and of these only eight were judged to be based on ‘strong’ empirical footings. The review finds strong evidence that the CCCQ measure is an internally consistent and reliable instrument with a confirmed factor structure. Other measurement properties require further research, in particular with respect to hypothesis testing where results using the measure may not accord with expectations. Further, the P-CAT has strong internal consistency and good content validity, with some limited evidence of test-retest reliability. However, five of the instruments reviewed have, at most, a single positively-rated measurement property to support their use. Furthermore, no instrument included in the review was the subject of successful measurement error, hypothesis testing or cross-cultural validity assessments. The review found no evidence that measures designed specifically for older adult services have superior measurement properties than their generic counterparts.

**Table 7:** Quality synthesis

Measure	Internal consistency	Reliability	Measurement error	Content validity	Structural validity	Hypothesis testing	Cross-cultural
*Specific measures*							
ICI	?	?		?	++	--	?
P-CAT	+++	+		++	+/−	?	
PCFC	+++			?	?		
PCHC	?			+++	++		
PDC	++			++	+		
‘Untitled’	?	?		?			
*Generic measures*							
PCQ (Staff version)	+++	-		?	++	?	?
PCQ (Patient version)	?	+		?		?	?
MPOCA	?	+		?		?	
CCCQ	+++	+++	?	?	+++	-	?
ICS-N	+				?		
CCRQ	?	++		+++		?	

Empty cells indicate the property was not been assessed in any reference for that measure. ‘+++’(‘---‘) indicates that ‘strong’ evidence supports a positive (negative) measurement property for that instrument; ‘++’(‘—‘) indicates ‘moderate’ evidence; ‘+’(‘-‘) indicates ‘limited’ evidence; ‘+/−‘indicates conflicting evidence; and ‘?’ indicates that only studies of poor quality were available or could not be determined

## Discussion

It has been argued that efforts to objectively measure person-centred care have not matched its rapid promotion amongst health service priorities [8]. Researchers seeking to evaluate interventions against these standards, and managers aiming to monitor and improve quality, have a limited evidence-base to support their choice of measurement instruments. Dow et al. [48] developed their own measure after a literature review found “no previously published measures of person-centered care in health settings” (p1066) was suited to their research in old age psychiatric services. No systematic review has hitherto been conducted and no formal quality appraisal has been undertaken. The present review aimed to fill this gap.

Eleven instruments were identified, spanning both general and gerontological nursing, rehabilitation and occupational therapy, and palliative care. However, the breadth and methodological quality of research underpinning these measures was generally poor, and none can be recommended without significant reservations. Two measures, the P-CAT and CCCQ, have been subject of the most attempts to test measurement properties. The former was designed for completion by staff in long-term care facilities to self-assess the person-centred quality of their service. However, the dimensionality of the instrument is subject to some uncertainty, with an unstable two or three factor solution in different language versions. Further development work and confirmatory factor analyses would bolster the measure. The CCCQ, by contrast, is a unidimensional measure developed primarily for home care services. The items were formulated directly from quotations from service user interviews, although these were mostly younger adults, and none aged over sixty [50]. The measure has been subject to the most rigorous testing of all the instruments, although there are doubts over its construct validity since it failed to conform to hypotheses tested in one study. There is some evidence that the items may have proved challenging for an elderly population to answer, and the authors have recommended the development of instruments using items tailored to the experiences and abilities of the particular client group being researched [42]. Cognitive interviewing is one method for rigorously testing the applicability and comprehension of instruments prior to wider piloting, and was adopted by only one reference in this review [49].

Quality appraisal was attempted for seven measurement properties across the 12 instruments (treating the two PCQ versions separately), permitting 84 possible synthesis results. Yet many measurement properties had not been successfully tested in any study meeting the eligibility criteria. Of the 51 populated cells, the review found only eight were supported by evidence rated as ‘strong’, with many ‘undetermined’ results due to poor methodological quality. The evidence-base would be better served by more studies of higher quality, even if that is at the expense of fewer measurement properties being investigated, at least in early development. For example, efforts to translate existing instruments into a multitude of new languages appear wasteful if the instrument has yet to demonstrate solid validity and reliability in its original language. Furthermore, using exploratory analysis with a fledgling instrument, and applying a *post-hoc* rationalisation of how results support validity, should be avoided. Not all such studies explicitly sought to formally establish a measurement property and might have been excluded from the review. Regardless, by not specifying clear hypotheses *a priori*, they did not achieve a good rating. Use of modern toolkits, such as COSMIN, may assist researchers in reaching the standards expected in modern measurement studies.

In common with Edvardsson et al. [32], this review also finds a notable lack of service user or carer perspectives in selecting items for inclusion in questionnaires. In addition to being poor practice, it is ironically incompatible with person-centredness. Researchers have also shied away from supplying evidence of measurement error, and its related concept, ‘minimal important change’ [26]. It is essential to understand what the smallest change is in any given measure that has meaning and value to service users. This can then be compared to estimated error within the measure, and it can then be determined if meaningful change is within or beyond what can be reliably detected. Thus, greater consideration to service user perspectives in the development and testing of future instruments is required.

The review strived to include measures of person-centredness in care, but given definitional ambiguities in the construct, the instruments synthesised are diverse in their content and intended application. Three domains of person-centredness - used as an operational definition for this review - were well represented in the measures; however it is evident that some implicitly used wider interpretations. Examples include an extension of person-centredness into patient perceptions of personal safety and physical comfort. There is nothing inherently problematic in this as long as researchers’ own interpretations of person-centredness are clearly articulated when developing their instruments, and, of course, that those using the questionnaires are alert to this. That said, instrument developers should at least ensure that the items reflect their intent: to achieve a high rating for content validity, the COSMIN framework demands that studies provide evidence that they assessed how comprehensively the items spanned their construct, and this was rarely demonstrated.

Other methodological considerations of this review should be borne in mind when interpreting the results. First, the COSMIN framework is relatively new, and its implementation is not without some travails. Some rating decisions remain a matter of significant judgement as to what constitutes violations of particular standards. Examples include whether hypothesis testing was guided by “adequate” or “poor” descriptions of comparison instruments, without a guide to expectations. Future development and testing of the COSMIN framework would be welcome. Second, the review is limited by its focus on questionnaire-based instruments. Observation-based measures, such as Dementia Care Mapping [51], may be suited to some research circumstances and could form a basis for criterion validity assessments. Further, the review has restricted its focus to measures developed and/or tested in older adult services, though this is not to say that other measures are necessarily inappropriate. However, before using such instruments, it would be imperative to inspect and test their validity with older people using long-term care services.

## Conclusions

Person-centredness is now regarded as a central component of any high quality long-term care service for older people. However, those seeking to evaluate change and improve standards have limited evidence to support their choice of measurement instruments. This review aimed to identify, describe and critically appraise relevant measures. Eleven instruments were included. The review found that references testing measurement properties were generally of low methodological quality. Two measures (the P-CAT and CCCQ) stand-out as having been tested beyond the initial development stages, though concerns remain over the structural validity of the former, and construct validity of the latter. The review recommends closer attention to methodological quality in testing measurement properties, and greater inclusion of service users and families in item development and validation.

## Additional file

Additional file 1: PRISMA 2009 Checklist. “Person-centredness in the care of older adults: a systematic review of questionnaire-based scales and their measurement properties”. (DOC 62 kb)
